# A Ru(II)-Strained Complex with 2,9-Diphenyl-1,10-phenanthroline Ligand Induces Selective Photoactivatable Chemotherapeutic Activity on Human Alveolar Carcinoma Cells via Apoptosis

**DOI:** 10.3390/ph17010050

**Published:** 2023-12-28

**Authors:** Najwa Mansour, Stephanie Mehanna, Kikki Bodman-Smith, Costantine F. Daher, Rony S. Khnayzer

**Affiliations:** 1 Department of Natural Sciences, Lebanese American University, Chouran, Beirut 1102-2801, Lebanon; najwa.mansour@lau.edu.lb (N.M.); stephanie.mehanna@lau.edu.lb (S.M.); cdaher@lau.edu.lb (C.F.D.); 2 Department of Microbial and Cellular Sciences, Faculty of Health and Medical Sciences, University of Surrey, Guildford GU2 7XH, UK; k.bodman-smith@surrey.ac.uk

**Keywords:** ruthenium, anticancer, A549, photoactivatable chemotherapy, in vitro photochemotherapy

## Abstract

[Ru(bipy)_2_(dpphen)]Cl_2_ (where bipy = 2,2′-bipyridine and dpphen = 2,9-diphenyl-1,10-phenanthroline) (complex **1**) is a sterically strained compound that exhibits promising in vitro photocytotoxicity on an array of cell lines. Since lung adenocarcinoma cancer remains the most common lung cancer and the leading cause of cancer deaths, the current study aims to evaluate the plausible effect and uptake of complex **1** on human alveolar carcinoma cells (A549) and mesenchymal stem cells (MSC), and assess its cytotoxicity in vitro while considering its effect on cell morphology, membrane integrity and DNA damage. MSC and A549 cells showed similar rates of complex **1** uptake with a plateau at 12 h. Upon photoactivation, complex **1** exhibited selective, potent anticancer activity against A549 cells with phototoxicity index (PI) values of 16, 25 and 39 at 24, 48 and 72 h, respectively. This effect was accompanied by a significant increase in A549-cell rounding and detachment, loss of membrane integrity and DNA damage. Flow cytometry experiments confirmed that A549 cells undergo apoptosis when treated with complex **1** followed by photoactivation. In conclusion, this present study suggests that complex **1** might be a promising candidate for photochemotherapy with photoproducts that possess selective anticancer effects in vitro. These results are encouraging to probe the potential activity of this complex in vivo.

## 1. Introduction

Over the years, ruthenium complexes have gained considerable interest in cancer therapy as alternatives for platinum drugs since they are believed to possess greater cytotoxicity and selectivity to cancer cells, thus eliciting fewer side effects. It is, in fact, speculated that the hypoxic environment of tumor cells may reduce and hence activate some Ru(III) prodrugs that are originally inert [1,2,3]. Additionally, cancer cells overexpress the transferrin receptors where Ru complexes can preferentially bind and enter the cells through endocytosis, conferring more selectivity towards cancer [4,5,6,7]. Previous studies have also suggested that some Ru complexes may reduce cancer metastasis by preventing cells from detaching, migrating and re-adhering to distant locations [8,9,10,11].

The Ru(III) complex KP1019 and its sodium analog KP1339 (KP1019 = trans-[tetrachlorobis(1H-indazole)ruthenate(III)], as well as NAMI-A (trans-[tetrachloro(DMSO)(imidazole)ruthenate(III)]) were the first ruthenium drugs that demonstrated in vitro cytotoxicity and selectivity towards solid tumors. They were shown to be effective in inducing cell death through apoptosis by blocking the cell cycle at the G2 phase [12]. NAMI-A was also shown to reduce cancer metastasis by inhibiting matrix metallopeptidases and increasing the extracellular matrix around tumors [13,14]. When they reached clinical trials, these drugs showed adverse side effects, including hepatotoxicity, neutropenia and gastrointestinal issues [13,14], thus discontinuing further research on the potential application of these drugs in cancer therapy [15]. Additional ruthenium complexes have recently shown promising chemotherapeutic activity and are currently under clinical trials [16,17,18].

Photoactivated chemotherapy (PACT) is a type of anticancer treatment that provides a turn-on potency effect in malignant cells through the targeted light activation of photosensitizing agents (PSs) or photoactivatable compounds [19]. The investigation of different photoactivatable Ru drugs as potential chemotherapeutic agents has been on the rise in the last few years [20,21,22,23,24,25,26]. Ideally, the photoactivatable Ru complex is administered as a prodrug and is photodynamically activated with light exclusively at the targeted cancer tissues [27,28,29]. Upon light activation, the prodrug may undergo ligand ejection, DNA crosslinking or photocaging effects [29,30,31,32], leading to cell death through variable mechanisms. In a recent study, a PDT ruthenium complex, the rGO-Ru-PEG complex with phosphorescent polyethylene glycol (PEG) and a reduced nanographene oxide (rGO) was shown to induce apoptosis, primarily via generation of reactive oxygen species (ROS) [33]. In another study, a light-activated Ru(II) complex showed an inducible DNA-binding affinity and obstruction in cellular DNA phase separation, suggesting its potential use as a chemotherapeutic drug [34].

To the best of our knowledge, none of the published photoactivatable Ru complexes that were tested in vitro or in vivo have reached clinical trials.

Improving the selectivity of chemotherapeutic agents constitutes a major challenge in anticancer drug development. Ideally, a selective drug must be able to exclude normal proliferating cells by targeting specific hallmarks of malignant cells. The mode of cellular uptake is one of the determinants of selectivity; however, few studies include a thorough investigation of the transport mechanisms across cancer cells, especially with the new generation of cytotoxic ruthenium complexes.

**Figure 1:**
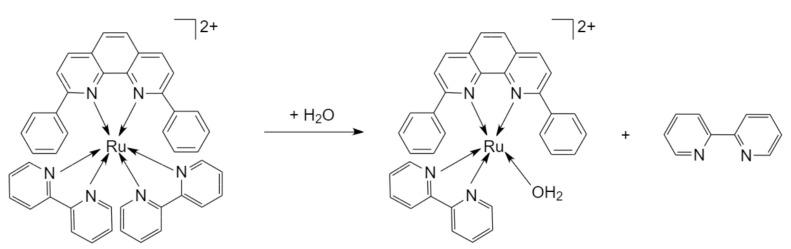
The photo-induced chemical conversion of [Ru(bipy)_2_(dpphen)]Cl_2_ (complex **1**) in water. Upon irradiation in water, the complex releases a 2,2′-bipyridine (bipy) ligand. The resulting Ru photoproduct is postulated to have an aqua and 2,9-diphenyl-1,10-phenanthroline (dpphen) ligand. The possibility of proton loss from the photoproduct was observed as yielding the singly charged cyclometallated Ru complex.

The synthesis, purification and characterization of a photoactivatable Ru(II) complex [Ru(bipy)_2_(dpphen)]Cl_2_ (where bipy = 2,2′-bipyridine and dpphen = 2,9-diphenyl-1,10-phenanthroline) (complex **1**) has been previously reported by our group along with cytotoxicity screening against different cancer cell lines [33]. Upon irradiation in water, the complex ejects a bipy ligand and becomes aquated (Figure 1).

The phototoxicity index (PI) values reported ranged between 39.2 and >100 folds, thus suggesting increased anticancer activity of complex **1** upon photoactivation. The present study aims at quantifying the cellular uptake of complex **1** in human alveolar carcinoma cells (A549) and mesenchymal stem cells (MSC), elucidating the possible mode of uptake involved as well as assessing the cytotoxic activity of complex **1** in vitro. The effect of the drug on cell morphology, membrane integrity, DNA and cell viability was assessed, and the toxicity against non-cancer cells was investigated to probe the selectivity of the drug against A549 cancer cells.

## 2. Results

### 2.1. Complex ***1*** Uptake by MSC and A549 Cells

#### 2.1.1. Quantification of Cellular Uptake by LC-MS/MS

**Figure 2:**
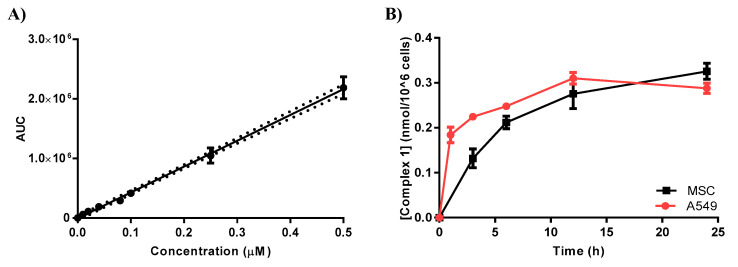
Uptake of complex **1** by MSC and A549 cells. (**A**) The calibration curve was established using LC-MS/MS, where the area under the curve (AUC) was plotted against concentration (µM). (**B**) A549 and MSC cells were exposed to complex **1** (3 µM), and the uptake was measured over various time intervals. The reported values were estimated based on the calibration curve and represent the mean ± SEM from three separate experiments.

A calibration curve was established using a serial dilution of complex **1** in cells extracted with acetonitrile. The cellular uptake was calculated using the best-fit value and interpolated at a 95% confidence interval (Figure 2A) in accordance with a published method [34]. Both MSC and A549 cells were treated with complex **1** (3 µM) and then incubated for varying durations (0, 1, 3, 6, 12 and 24 h), with a subsequent analysis of cellular uptake via LC-MS/MS. It was observed that complex **1** uptake exhibited a significant increase within the initial hour, reaching its peak after a 12 h incubation period. The maximum detected uptake concentration for both cell lines was approximately 0.3 nmol/10^6^ cells, as shown in Figure 2B.

#### 2.1.2. Evaluating the Mode of Cellular Uptake

To investigate the mechanism of cellular uptake of complex **1**, MSC and A549 cells were subjected to various conditions prior to LC-MS/MS analysis. The uptake of complex **1** in both cell lines was not significantly affected by the addition of KCl solution, which usually alters the membrane potential. This suggests that passive or facilitated diffusion mechanisms were not involved in the uptake [35].

**Figure 3:**
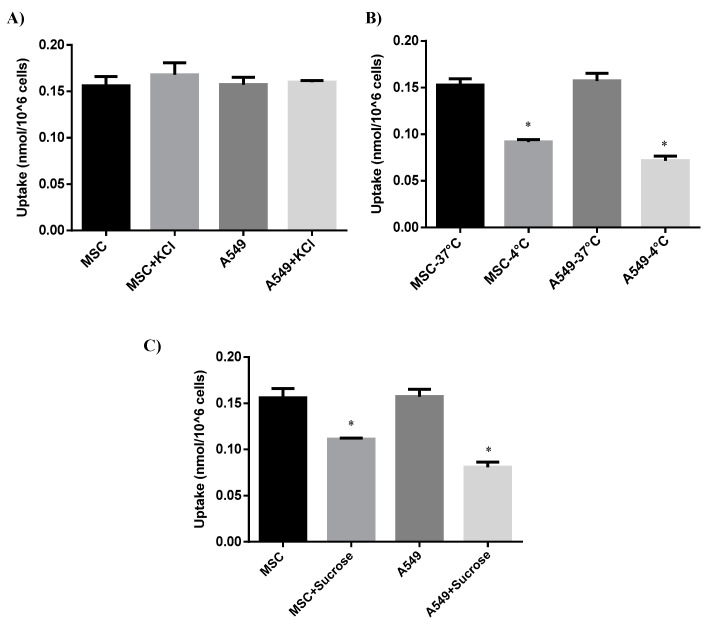
The mechanism of complex **1** uptake by MSC and A549 cells. (**A**) Cells were exposed to complex **1** (3 µM) in the presence or absence of KCl and then incubated for 2 h at 37 °C. (**B**) Cells were treated with complex **1** (3 µM) and then incubated in DMEM for 2 h at either 4 °C or 37 °C. (**C**) Cells were treated with complex **1** (3 µM) with or without 0.45 M sucrose and incubated for 2 h at 37 °C. Cellular uptake was evaluated using LC-MS/MS, and the values provided represent the mean ± SEM from three distinct experiments. * Indicates a significant difference (*p* < 0.05) compared to the control.

Furthermore, both cell lines were treated with complex **1** and incubated at either 4 °C or 37 °C to determine whether the uptake was mediated by endocytosis or carrier-mediated active transport [36,37]. The findings demonstrated that lower temperatures, which interfere with energy-dependent transport, significantly decreased the complex **1** uptake in both cell lines (*p* < 0.05). Then, to distinguish between active transport via carrier-mediated uptake and endocytosis, a hypertonic sucrose solution was added. The latter significantly hindered complex **1** uptake in both MSC and A549 cells (*p* < 0.05), as demonstrated in Figure 3C, pointing to a potential energy-dependent method of entry via endocytosis [36]. The treatment conditions and findings above are summarized in Table 1.

### 2.2. Effect of Complex ***1*** on the Survival of A549 and MSCs

**Figure 4:**
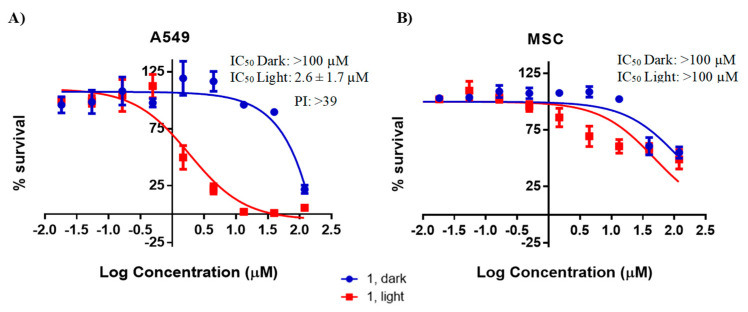
The dose–response relationship for cytotoxicity of complex **1** on A549 cells (**A**) and MSC cells (**B**) was assessed after 72 h of treatment, both with and without light activation. The values represent the mean ± SEM derived from three separate experiments.

Complex **1** was administered to A549 cells for 24, 48 and 72 h, both with and without light activation (as depicted in Figure S1), and the WST-1 kit was used to assess cell viability. The phototoxicity index (PI), which is the ratio of the IC_50_ of the drug without light activation to the IC_50_ of the drug after light activation, was calculated and is presented in Table 2. In the absence of light activation, the IC_50_ values exceeded 100 µM for both cell lines. However, upon light activation, cytotoxicity exhibited a time-dependent increase, particularly in the A549 cell line, with a maximum PI value exceeding 39 folds observed at 72 h post-treatment. As a result, all subsequent experiments, including the evaluation of MSC cell survival (as shown in Figure 4), were conducted after a 72 h incubation period. Furthermore, the data indicated that with light activation, complex **1** demonstrated significantly greater cytotoxicity against A549 cells (IC_50_ = 2.6 ± 1.7 µM) as compared to MSC cells (IC_50_ > 100 µM) (Figure 4).

### 2.3. Effect of Complex ***1*** on A549 Cell Morphology and Detachment

**Figure 5:**
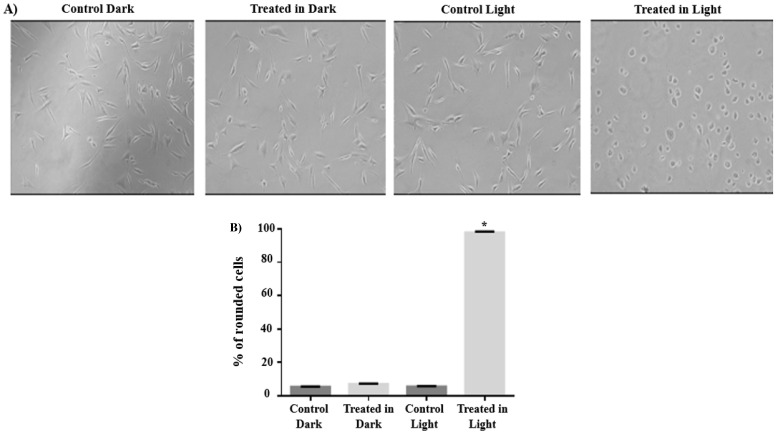
Morphological changes in A549 cells following a 72 h exposure to complex **1** at a concentration of 5 µM, either with (light) or without light activation (dark). (**A**) Images were captured to illustrate the morphological variations in cells under different conditions. (**B**) The percentage of cell rounding in A549 cells was quantified. Data analysis was performed by expressing % cell rounding using CASP software version 6.0. The mean ± SEM was computed based on three independent experiments, during which a total of ten randomly selected images from each experiment were observed under a microscope. * Indicates significant variations between the control and treated cells (*p* < 0.05).

A549 cells were exposed to complex **1** at a concentration of 5 µM, either with or without subsequent light activation, and then incubated for 72 h. Cells treated with complex **1** without light activation did not display any noticeable alterations in their morphology (as shown in Figures S2 and S3 and Figure 5). In contrast, upon irradiation, complex **1** led to an increase in cell rounding, indicating a significant change in cell morphology (as illustrated in Figure 5A,B).

### 2.4. Effect of Complex ***1*** on A549 Cell Membrane Integrity

**Figure 6:**
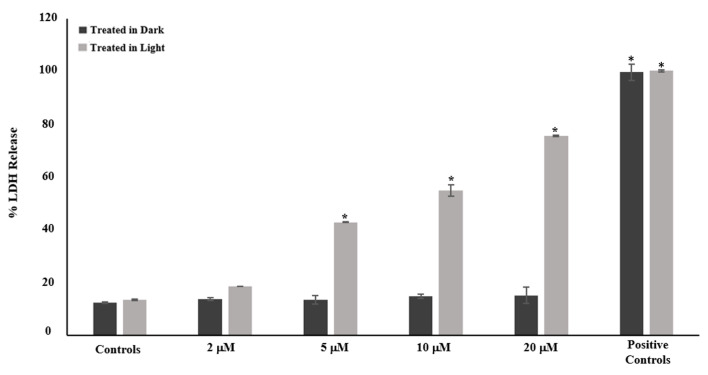
The percentage of LDH release from A549 cells following a 72 h exposure to varying concentrations of complex **1** (2, 5, 10 and 20 µM). The data are displayed as the mean ± SEM derived from three separate experiments. * Indicates significant variances between control cells and treated cells (*p* < 0.05).

Complex **1** was added to A549 cells at doses of 2, 5, 10 and 20 µM, followed by either light activation (light) or incubation in the dark. The LDH release was evaluated after 72 h, as shown in Figure 6. The greatest LDH release (100%) was seen in the positive control. Untreated cells, as well as cells treated with complex **1** without irradiation, did not exhibit an increase in LDH release. However, after being exposed to complex **1** and light, the cells showed a dose-dependent increase in LDH release. Following light activation, treatment with 20 µM of complex **1** caused a maximal loss of membrane integrity, with the LDH release reaching 75.7%. This aligns well with the concentration range associated with the lowest percentage of cell survival, as observed in Figure 4.

### 2.5. DNA-Damaging Effect of Complex ***1***

#### 2.5.1. Plasmid DNA Damage

**Figure 7:**
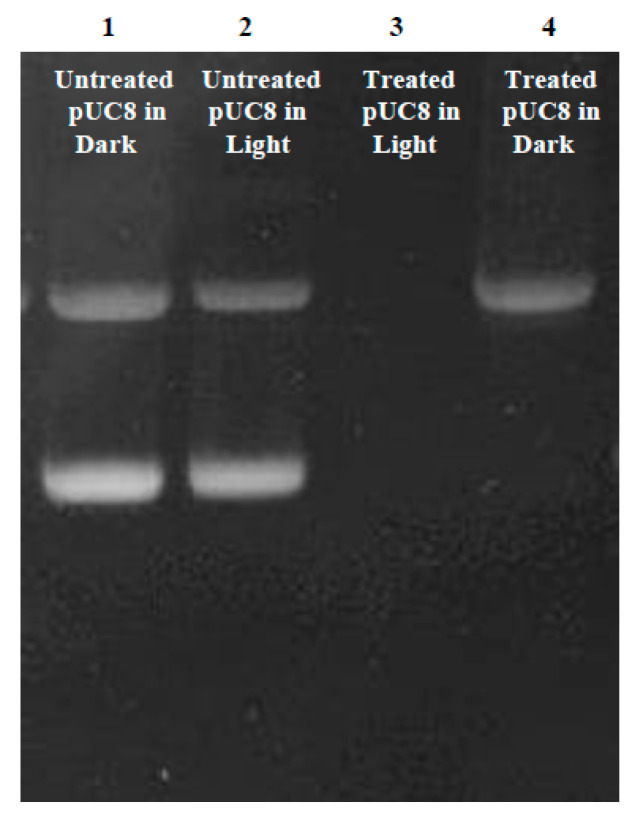
The effect of complex **1** on the pUC8 plasmid. The impact of complex **1** on the pUC8 plasmid was assessed through the following experimental conditions: pUC8 plasmid kept in the dark (lane 1), pUC8 plasmid exposed to light (lane 2), pUC8 plasmid mixed with 5 μM of complex **1** and subjected to light activation (lane 3), and pUC8 plasmid mixed with 5 μM of complex **1** without light activation (lane 4). After 12 h, gel electrophoresis was conducted, and the results were visualized and captured using the Bio-Rad ChemiDoc™ gel imaging system.

Complex **1** (5 μM) was combined with the pUC8 plasmid, and the DNA damage was evaluated, both with and without light activation, using 1% agarose gel electrophoresis. The results were visualized using the Bio-Rad ChemiDoc™ (Hercules, CA, USA) gel imaging system (Figure 7). Two bands could be seen in lanes 1 and 2, which represent the control, untreated pUC8 (in the dark or exposed to light). The pUC8 plasmid is represented by the bottom band in its supercoiled form (faster band) and the upper band in its relaxed state (slower band). With no light activation, the plasmidic DNA treated with complex **1** only displayed the upper band representing the pUC8 relaxed form (lane 4). However, there was no band seen in lane 3 where pUC8 was treated with complex **1** followed by irradiation. The EtBr signal was completely lost, most likely as a result of DNA cleavage or destruction.

#### 2.5.2. DNA Damage in A549 Cells

**Figure 8:**
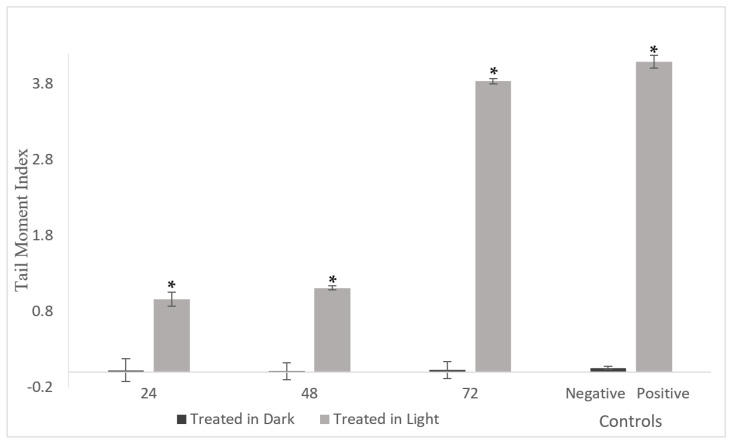
The effect of complex **1** on the A549 tail moment index after 24, 48 and 72 h of treatment (5 µM), both in the presence of light (light) and in the absence of light (dark). KMnO_4_ and PBS (25 µM) were used as positive and negative controls, respectively. The data are presented in bar graphs, showing the mean ± SEM derived from the analysis of 150 randomly selected images collected from three distinct experiments (50 images per experiment). * Indicates significant differences between the negative control and treated cells (*p* < 0.05).

Cells were exposed to complex **1** at a concentration of 5 µM and were either kept in the presence or absence of light activation. Subsequently, the DNA damage was evaluated at 24, 48 and 72 h. Using CASP software, measurements were obtained for head length, tail length, comet length, head DNA content, tail DNA content and tail moment (as detailed in Table S1). This analysis was performed on 50 randomly selected cells for each condition. Complex **1** treatment for 24, 48 and 72 h without light activation resulted in negligible DNA damage (low TMI). The complex did, however, significantly increase DNA damage (elevated TMIs), beginning as early as 24 h (as depicted in Figure 8).

### 2.6. Effect of Complex ***1*** on A549 Cell Death

**Figure 9:**
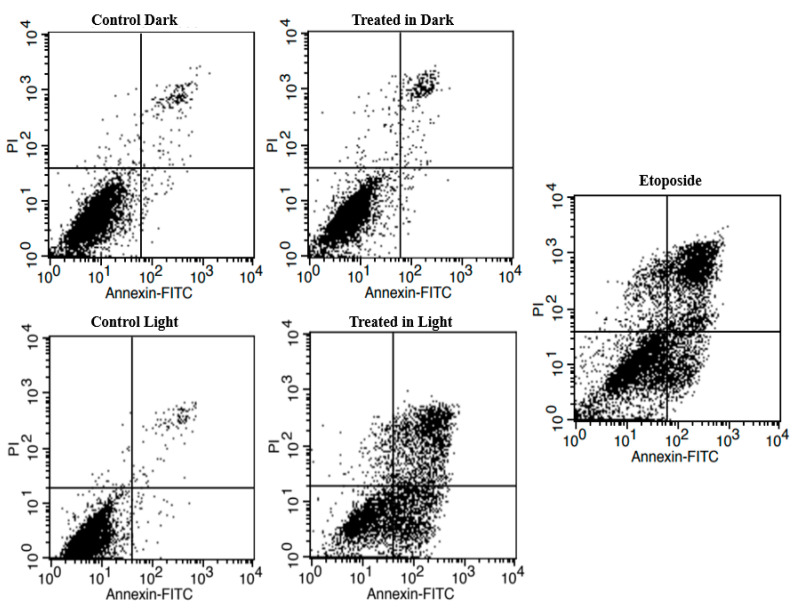
A549 cell survival upon treatment with complex **1** (5 μM) for 72 h using flow cytometry. Etoposide served as the positive control for apoptosis assessment, with results obtained after 48 h of incubation. The data are presented as bar graphs, indicating the mean ± SEM, which was computed from three distinct experiments. * Indicates significant differences between the negative control and treated cells (*p* < 0.05).

Complex **1** (5 μM) was added to A549 cells, and the latter were either exposed to light or left in the dark, followed by a 72 h incubation. The results obtained by flow cytometry indicated that there was no significant cell death observed in the control cells, whether they were exposed to light (94.2 ± 1.3% live cells) or not (93.6 ± 1.2% live cells), and in cells treated with complex **1** without light activation (92.7 ± 1.5% live cells) (as shown in Figure 9 and Table 2). However, it was observed that treatment with etoposide and complex **1** after light activation led to a significant increase in apoptosis (34.4 ± 1.7% and 62.5 ± 1.3% early apoptotic cells, respectively), with the percentages of live cells decreasing to 20.5 ± 2.0 and 10.8 ± 1.2%, respectively (Figure 9 and Table 3).

## 3. Discussion

Photoactivatable ruthenium complexes have shown interesting preliminary outcomes in targeted cancer therapy [27,29]. In this work, the uptake and cytotoxicity of the photoactivatable drug [Ru(bipy)_2_(dpphen)]Cl_2_ (complex **1**) was evaluated against normal MSC and A549 cancer cells, and its mechanism of action was investigated. Data showed that the cellular uptake of complex **1** by non-cancerous MSC and A549 human alveolar carcinoma cells started within the first hour of administration and increased over time, reaching a maximum after 12 h of incubation. This is in line with previous studies on other ruthenium-based anticancer drugs [26,37,38,39,40,41], and as a result, complex **1** was incubated for 12 h in all the experiments to allow for maximum drug uptake before light activation.

After determining the optimal timing for the maximal uptake of complex **1**, it became crucial to comprehend how this ruthenium drug enters cells for its practical development and application. Passive and facilitated diffusion, not reliant on energy, are the least prone to modification by structural analogs. Conversely, alterations in membrane fluidity can be influenced by factors like cholesterol depletion or low temperature. If the uptake occurs through passive or facilitated diffusion, manipulating the potassium concentration gradient can specifically affect membrane potential, thus influencing cellular uptake [35,42]. By exposing cells to an isotonic potassium buffer with a concentration akin to that within cells, the cell membrane potential can effectively be diminished to zero [43].

The results of the present study indicate that altering the membrane potential with isotonic potassium buffer incubation did not significantly affect the cellular uptake of complex **1**, ruling out passive and facilitated diffusion as primary uptake mechanisms. On the contrary, active transport, whether through carrier-mediated uptake or endocytosis, can be affected by variables like temperature (4 °C) or the introduction of ATP inhibitors [44,45]. To ascertain whether the active transport of complex **1** relies on carrier-mediated uptake and/or endocytosis, a hypertonic sucrose solution was introduced, impeding endocytosis by influencing endosomal activity [36]. The cellular uptake of complex **1** seemed to occur via an energy-dependent form of endocytosis, as it significantly decreased when incubated at low temperatures or with hypertonic sucrose. Previous research has proposed that ruthenium complexes, including KP1019, preferentially accumulate in cancer cells compared to non-cancer cells through transferrin receptor-mediated endocytosis, attributed to the heightened expression of these receptors on the surface of cancer cells [4,5,6,46].

The cytotoxicity of complex **1** was assessed on A549 and MSC cells. The toxicity was minimal without light activation (IC_50_ > 100 µM). However, upon light activation, the cytotoxicity was dose- and time-dependent. The PI values increased from 16 to 39 after 24 to 72 h of incubation, respectively, showing the potency of complex **1** photoproducts. Consequently, the remaining experiments were carried out for 72 h, which was consistent with previous studies on other ruthenium complexes [47,48,49,50,51,52]. We previously reported similar cytotoxic results for complex **1** against other cancer cell lines (B16-F10, Caco-2, MDA-MB-231 and HT29) with PI values ranging from 100 to 50.5 [37]. Romerosa and his coworkers evaluated the phototoxic effect of four Ru(II) complexes containing 1,3,5-triaza-7-phosphaafamantane (PTA) in A549 cells and reported increased cytotoxicity compared to dark conditions with PI values of up to 10.7 folds [51]. Similarly, our group reported high phototoxicity of the Ru(II) bipyridyl complex Ru(II)bipy_2_BC (where BC is bathocuproine), which exhibited a PI of 11.1 in A549 cells [52].

Despite similar uptake profiles, complex **1** exhibited lower photocytotoxicity against MSC at 72 h (PI~1-fold) when compared to A549 cells (PI > 39 folds). These findings suggest a potential selectivity of complex **1** towards cancer cells, possibly reducing the adverse effects associated with conventional chemotherapeutic drugs. The preferential accumulation of complex **1** in tumor cell mitochondria, leading to apoptosis, and the difference in repair capacity between cancer and non-cancer cells could be both possible explanations for the selective cytotoxicity [53,54,55]. Studies have revealed that non-cancerous cells exhibit a superior membrane repair capacity (98%) compared to cancer cells (81%) following electroporation-based therapies, indicating more effective recovery from plasma membrane permeabilization [56]. It was previously established that the photoactivation of complex **1** generates the aquated photoproduct [Ru(bipy)(dpphen)(H_2_O)]^+^ [37]. The positive charges of the latter might facilitate the interaction with cancer cell membranes, which are known to possess a highly negative potential [57], thus increasing selectivity.

Further studies are needed to assess the cytotoxicity of complex **1** on other normal cell lines as well as the accumulation in mitochondria.

The present study also showed that complex **1** treatment followed by light activation induced significant cell rounding and increased LDH release from A549 cells, indicating cell death through necrosis or apoptosis, as well as loss of membrane integrity. This is consistent with previous studies on other Ru complexes [58,59] as well as our previous study on the effect of complex **1** on triple-negative human breast adenocarcinoma cells (MDA-MB-231 cells) [38].

Preliminary screening for DNA damage using the plasmid pUC8 revealed that this plasmid exists in two forms: a supercoiled and a relaxed form. The latter is more spread out and has slower mobility [60,61]. Without photoactivation, complex **1** caused the disappearance of the supercoiled form. This suggests that complex **1** may have bound DNA and induced the formation of the relaxed form of the plasmid instead of the supercoiled form. Following light activation, complex **1** leads to the disappearance of both bands, suggesting a possible interaction with DNA, causing fragmentation. DNA fragmentation is often observed upon treatment of cancer cells with ruthenium-based drugs [62,63,64,65]. We have previously shown that irradiation of Ru(II)bipy_2_BC in pUC18 leads to the disappearance of the EtBr signal, suggesting DNA damage and fragmentation [52]. Romerosa and his colleagues showed that the Ru(II) bipyridyl complexes *cis*-[Ru(dcbpyH)_2_(PTAH)_2_]Cl_2_·3H_2_O (where dcbpy = 4,4′-dicarboxy-2,2′-bipyridine and PTA = 1,3,5-triaza-7-phosphaadamantane) as well as *trans*-[Ru(bpy)_2_(PTA)_2_](CF_3_SO_3_)_2_ unfold supercoiled DNA upon photoactivation [51]. Intercalation and induction of DNA damage by complex **1** upon light activation were further assessed using the comet assay [66,67,68]. DNA is normally supercoiled, confined in the nucleoid region, and does not migrate much upon electrophoresis. In DNA-damaged cells, the DNA is unwounded, and this migrates rapidly out of the nucleoid, which translates as comet tails on gel electrophoresis [67,69]. A549 cells treated for 24 h showed an increase in TMI, indicating DNA damage and fragmentation. Apoptosis, characterized by cellular morphological changes and DNA fragmentation, was further confirmed by flow cytometry [10,70].

Our group has previously reported that upon photoactivation, complex **1** dissociates into an aquated photoproduct [Ru(bipy)(dpphen)(H_2_O)]^+^ and bipy ligand. The dissociating bipy ligand has been shown to be non-cytotoxic [37]. We have also reported that drug-free irradiation for a short duration is not toxic, both in vitro and in vivo [71]. All these results combined indicate that the photocytotoxicity of complex **1** is induced by the Ru photoproduct [Ru(bipy)(dpphen)(H_2_O)]^+^. The latter potentially triggers A549-cell rounding and detachment, loss of membrane integrity, and DNA damage, leading to apoptotic cell death.

The suggested in vitro activity of complex **1** in this study, as well as previous reports on other cell lines [37,38], encourage further investigation of the drug in vivo. The therapeutic application is, however, limited to superficial tumor models owing to the low penetrability of blue wavelengths [72].

## 4. Materials and Methods

### 4.1. Chemicals and Reagents

Dulbecco’s Modified Eagle Medium (DMEM), phosphate bovine serum (PBS), trypsin (1x) and penicillin-streptomycin were purchased from Biosera (Nuaille, France). Fetal bovine serum (FBS) was from Gibco^®^ (Waltham, MA, USA). The LC-MS grade solvents were purchased from Fisher Chemical. WST-1 reagent, the Comet assay and the CytoTox 96^®^ Non-Radioactive Cytotoxicity Assay kits were from Roche (Ludwigsburg, Germany), Trevigen (Gaithersburg, MD, USA) and Promega (Madison, WI, USA), respectively.

### 4.2. Synthesis of Complex ***1***

The synthesis, purification and characterization of complex **1** were previously described by our group [37].

### 4.3. Cell Lines and Culture

A549 cells were purchased from the American Type Culture Collection (ATCC, Manassas, VA, USA). MSCs were isolated from rat bone marrow as previously described [52], and all procedures complied with the Guide for the Care and Use of Laboratory Animals [73,74]. Both cancer cells and MSCs were maintained in DMEM at 37 °C with 5% CO_2_ [75].

### 4.4. Quantification of Cellular Uptake of Complex ***1***

The cellular uptake of complex **1** by A549 or the MSCs was quantified as previously reported [53,76]. Briefly, cells were seeded at a final concentration of 1.2 × 10^5^ cells/mL and allowed to adhere overnight at 37 °C/5% CO_2_. Complex **1** (3 µM) was then added to the cells, followed by incubation for 0, 1, 3, 6, 12 or 24 h. After removing the medium and washing with PBS, the cells were harvested by scraping in acetonitrile, homogenized and centrifuged for 10 min at 16,000× *g*. Supernatants were analyzed by LC-MS/MS using external standards. The instrument parameters are described in the Supplementary File, and the LC-MS/MS conditions are presented in Table S2. Data were reported as the mean concentration of complex **1** ± SEM of three different experiments and expressed in nmol/10^6^ cells.

### 4.5. Evaluating the Modes of Cellular Uptake of Complex ***1***

To evaluate the mode of cellular uptake, A549 or the MSCs were plated at a final concentration of 1.2 × 10^5^ cells/mL and were allowed to adhere overnight at 37 °C/5% CO_2_. Complex **1** (3 µM) was then added to the cells and the plates were incubated under three different conditions: (1), at 4 or 37 °C for 2 h; (2) in the DMEM medium supplemented with 0.45 M sucrose at 37 °C for 2 h; (3) in PBS buffer containing Na_2_HPO_4_ (30 mM), KH_2_PO_4_ (1.76 mM) and glucose (0.1%) at a pH of 7.4 and supplemented with KCl (2.7 mM) at 37 °C for 2 h. The cells were then lysed, and the supernatants were analyzed by LC-MS/MS using external standards. The results were reported as the mean (concentration of complex **1**) ± SEM of three different experiments and expressed as nmol/10^6^ cells [77,78].

### 4.6. Irradiation Conditions

For all relevant biological experiments, photoactivation was accomplished for 40 min using a home-built 460 nm LED light (Engin LZ4-40B208-000; 100 mW cm^−2^) operated at 50% of its full power. Further, 96- or 6-well plates were placed at a fixed distance from the light source (1 cm). The photochemistry of the complex has been previously reported [37].

### 4.7. Cell Viability Assay

A549 or the MCSs were seeded in 96-well plates (10^4^ cells/well), and then complex **1** was added in 3-fold dilutions starting at 120 µM. The plates were incubated for 12 h, after which they were exposed to light, where applicable [79]. WST-1 reagent (Roche©) was used to assess cell viability at 24, 48 or 72 h for the A549 cells and at 72 h for the MSCs after photoactivation. Absorbance was measured at 450 nm using a Thermo Scientific Multiskan^®^ (Waltham, MA, USA) FC photometer [37]. The samples were run in triplicates, and each experiment was repeated thrice.

### 4.8. Cell Rounding Assay

A549 cells (2 × 10^4^ cells/mL) were incubated in 6-well plates and then treated with complex **1** (5 µM) for 12 h. Control cells were left untreated. The plates were then irradiated for 40 min or kept in the dark, followed by a 12 h incubation. All conditions were prepared in triplicates, and the experiment was repeated thrice. An inverted light microscope was used to take a total of 10 images per well at 10× magnification. Cell detachment and rounding were then quantified as previously described [58].

### 4.9. Cell Membrane Integrity Assay

The CytoTox 96^®^ Non-Radioactive Cytotoxicity Assay kit (Promega-G1780) was used to evaluate membrane integrity. Briefly, confluent A549 cells (10^4^ cells/well) were incubated with 2, 5, 10 or 20 µM of complex **1** for 12 h at 37 °C and then activated with light for 40 min or kept in the dark. After a 12 h incubation, the plates were assessed for the % LDH release as previously described [38]. The samples were analyzed in triplicates, and each experiment was repeated thrice.

### 4.10. Plasmid DNA Precipitation Assay

The assay was performed using pUC8 plasmid (Edvotek, Washington, DC, USA), as previously described [52]. Briefly, the plasmid was mixed with potassium phosphate buffer (10 mM, pH 7.4) and then incubated with complex **1** (5 μM) or otherwise left untreated. DNA suspensions were irradiated or left in the dark, and after a 12 h incubation, electrophoresis using a 2% agarose gel containing 0.1 mg/mL of ethidium bromide was performed in the presence of circular pUC8 as a negative control. The gel was visualized using the Bio-Rad ChemiDoc™ gel imaging system.

### 4.11. Comet Assay

Damage was assessed using the Comet assay kit from Trevigen (Gaithersburg, MD, USA), as previously described [38]. In brief, A549 cells (10^5^ cells/well) were incubated with complex **1** (5 µM) or otherwise left untreated. Light activation was performed as above, and the control cells were kept in the dark. After a 72 h incubation, DNA damage and comet tails were measured in the presence of a positive control (cells treated with 25 µM of KMnO_4_) and a negative control (cells treated with PBS). A total of 50 randomly selected cells were evaluated and scored for tail intensity using the Comet Analysis Software Package (CASP). The tail moment index (TMI) was calculated by multiplying the tail DNA content of cells by the tail length and dividing by 1000 [80,81].

### 4.12. Flow Cytometry Assay

To assess the apoptotic effect of complex **1** on A549 cells, the latter were stained with propidium iodide (PI) and Annexin V-fluorescin Isothiocyanate (FITC) (Abcam 14,083) and assessed by flow cytometry. Briefly, the cells were plated at a final concentration of 2.5 × 10^4^ cells/well and then incubated with complex **1** (5 μM) for 12 h or otherwise left untreated. The plates were either irradiated as mentioned before or kept in the dark, and after a 72 h incubation, both floating and attached cells were retrieved by centrifugation at 210 g for 5 min at 4 °C. Pellets were suspended in binding buffer (0.5 mL) containing propidium iodide (PI, 5 μL) and/or Annexin V-fluorescin Isothiocyanate (5 μL) for 5–10 min. Analysis was performed using the FACSCalibur flow cytometer at an excitation wavelength (Ex) of 488 nm and an emission wavelength (Em) of 530 nm. Etoposide was used as an apoptotic control after a 48 h treatment [77,78].

### 4.13. Statistical Analysis

Data are reported as the mean ± SEM from three different experiments. The results were analyzed by one-way analysis of variance (ANOVA) on GraphPad Prism software (Version 7.0). Differences in values were considered a statistically significant difference if *p* < 0.05 (computed by Tukey’s multiple comparisons test).

## 5. Conclusions

The cellular uptake of complex **1** was shown to occur via endocytosis, with a maximum intracellular concentration reached at 12 h of incubation. Complex **1** demonstrated promising anticancer activity against A549 cell lines upon photoactivation, resulting in cell rounding and detachment, loss of membrane integrity and DNA damage. Despite the similar cellular accumulation in both A549 and MSCs, the photoactivated complex **1** showed potential selectivity towards cancer cells in vitro. Importantly, cell death was found to be through apoptosis. These results suggest that complex **1** may serve as a potential photochemotherapeutic agent. Given the favorable in vitro results, research is currently underway to assess the activity of complex **1** photoproducts in vivo.

## Figures and Tables

**Table 1:** Conditions and findings used to assess the modes of cellular uptake for complex **1** using LC-MS/MS.

Conditions	Treatment	Findings
**1**	Replace DMEM with special PBS buffer supplemented with KCl (2.7 mM) and add 3 µM of complex **1** (at 37 ℃)	No change → Uptake is not by passive nor facilitated diffusion
**2**	Replace DMEM with fresh DMEM and add 3 µM of complex **1** (at 4 or 37 °C)	Significant decrease at 37 °C → Uptake is possibly via an active (energy-dependent) mode of entry
**3**	Replace DMEM with fresh DMEM supplemented with 0.45 M sucrose and add 3 µM of complex **1** (at 37 °C)	Significant decrease → Uptake is via endocytosis

**Table 2:** Cytotoxic effects of complex **1** on A549 cells after 24, 48 and 72 h of treatment and MSCs after 72 h, both with (light) and without light activation (dark). The data are presented as IC50 values, and the phototoxicity index (PI) is calculated as the ratio of the IC50 in the dark to the IC50 in the presence of light.

IC_50_—Complex **1**			
	24 h	48 h	72 h
A549	Dark: >100 ^a^ Light: 6.2 ^b^ ± 0.1 PI: >16	Dark: >100 ^a^ Light: 4.0 ^c^ ± 0.1 PI: >25	* Dark: >100 ^a^ Light: 2.6 ^c^ ± 1.7 PI: >39
MSCs			Dark: >100 Light: >100

Values with different superscript letters indicate statistically significant differences (*p* < 0.05), and all data represent the mean ± SEM derived from three separate experiments, each conducted in triplicate. * Previously published data [37].

**Table 3:** Percentage of live, early apoptotic, late apoptotic and necrotic A549 cells after treatment with 5 µM complex **1**.

Conditions	Live Cells	Early Apoptotic	Late Apoptotic	Necrotic Cells
Control Dark	93.6 ± 1.2	2.2 ± 0.89	2.5 ± 1.3	1.8 ± 0.3
Treated in Dark	92.7 ± 1.5	2.4 ± 1.7	2.4 ± 1.9	2.5 ± 0.36
Control Light	94.2 ± 1.3	2.2 ± 1.6	2.5 ± 1.2	1.1 ± 1.7
Treated in Light	10.8 ± 1.2 *	62.5 ± 1.3 *	21.3 ± 1.6 *	5.5 ± 2.9
Etoposide, 40 μM	20.5 ± 2.0 *	34.4 ± 1.7 *	40.8 ± 0.72 *	4.4 ± 1.3

* Denotes significant difference compared to the control (untreated cells), as measured by one-way ANOVA, and values present the mean ± SEM calculated from three independent experiments.

## Data Availability

Data are contained in the article and Supplementary Materials.

## Supplementary Materials

The following supporting information can be downloaded at: https://www.mdpi.com/article/10.3390/ph17010050/s1, Figure S1. Complex **1** cytotoxicity effect on A459 cells after 24, 48 and 72 h with or without light activation; Figure S2. Morphological changes and cell rounding on A549 cells upon treatment with different concentrations of complex **1** after 24 h; Figure S3. Morphological changes and cell rounding on A549 cells upon treatment with 5 µM of complex **1** for 24, 48 and 72 h; Table S1. DNA damage in A549 cells exposed to 5 µM of complex **1,** as measured by the comet assay after 24, 48 and 72 h post-exposure; Table S2. LC-MS/MS conditions used for the detection of Complex **1**.
