# Supplementary material for: A Ru(II)-Strained Complex with 2,9-Diphenyl-1,10-phenanthroline Ligand Induces Selective Photoactivatable Chemotherapeutic Activity on Human Alveolar Carcinoma Cells via Apoptosis
Source: Pharmaceuticals (Basel). 2023 Dec 28;17(1):50. doi: 10.3390/ph17010050 (PMC10819265; doi:10.3390/ph17010050)
Supplement: Supplementary file 1 [file pharmaceuticals-17-00050-s001.zip › pharmaceuticals-2669467-supplementary.pdf]

Supplementary Information

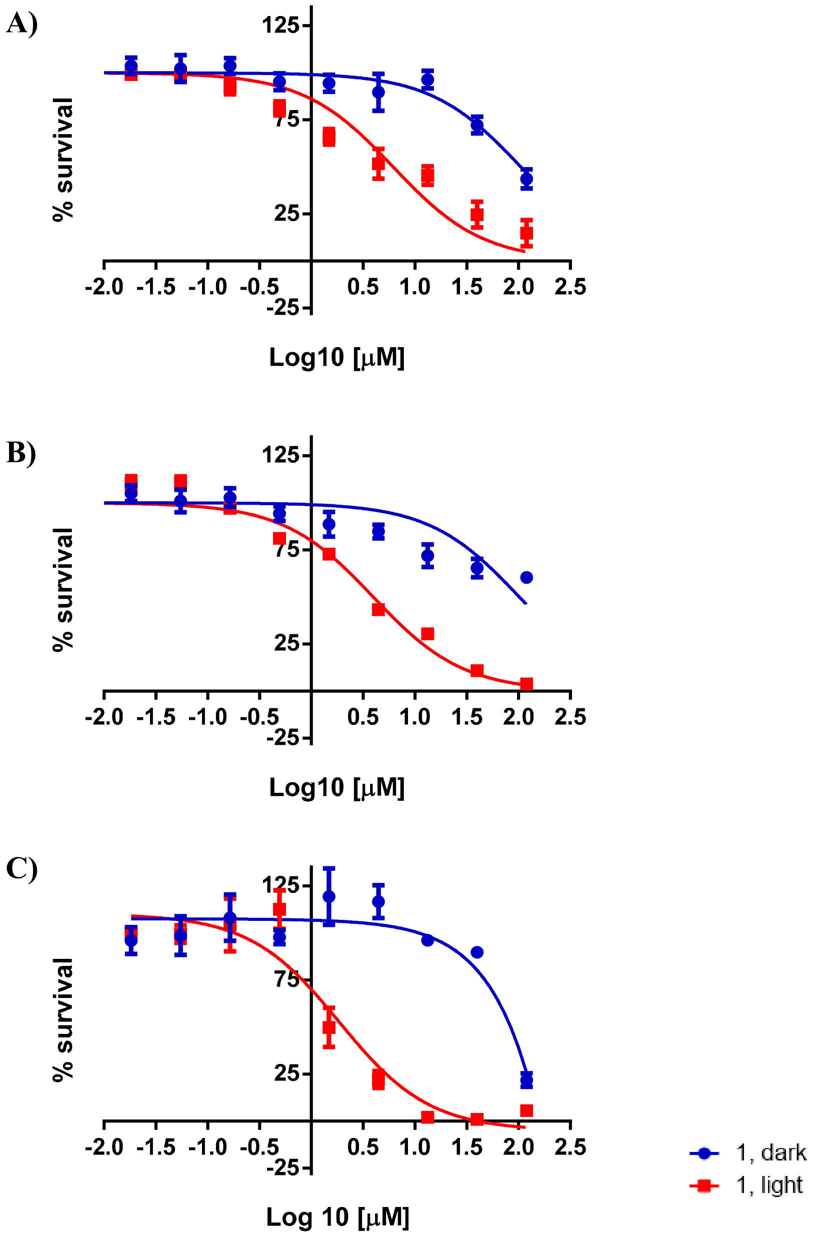

Figure S1. Complex 1 Cytotoxicity effect on A459 cells after 24, 48 and 72h with or without light activation. Treatment with complex **1** was completed using 3-fold dilutions starting with a highest concentration of 120 μM. Cells were then incubated with different concentrations of complex **1** in both dark and light conditions for 24, 48 or 72 h and cell viability was quantified. Data obtained were expressed as the percentage of surviving cells as a function of log (concentration) of complex **1**. The mean ± SEM values were calculated from three separate experiments, each performed in triplicate. Complex **1** showed an increase in cytotoxicity between dark and light conditions with longer incubation time.

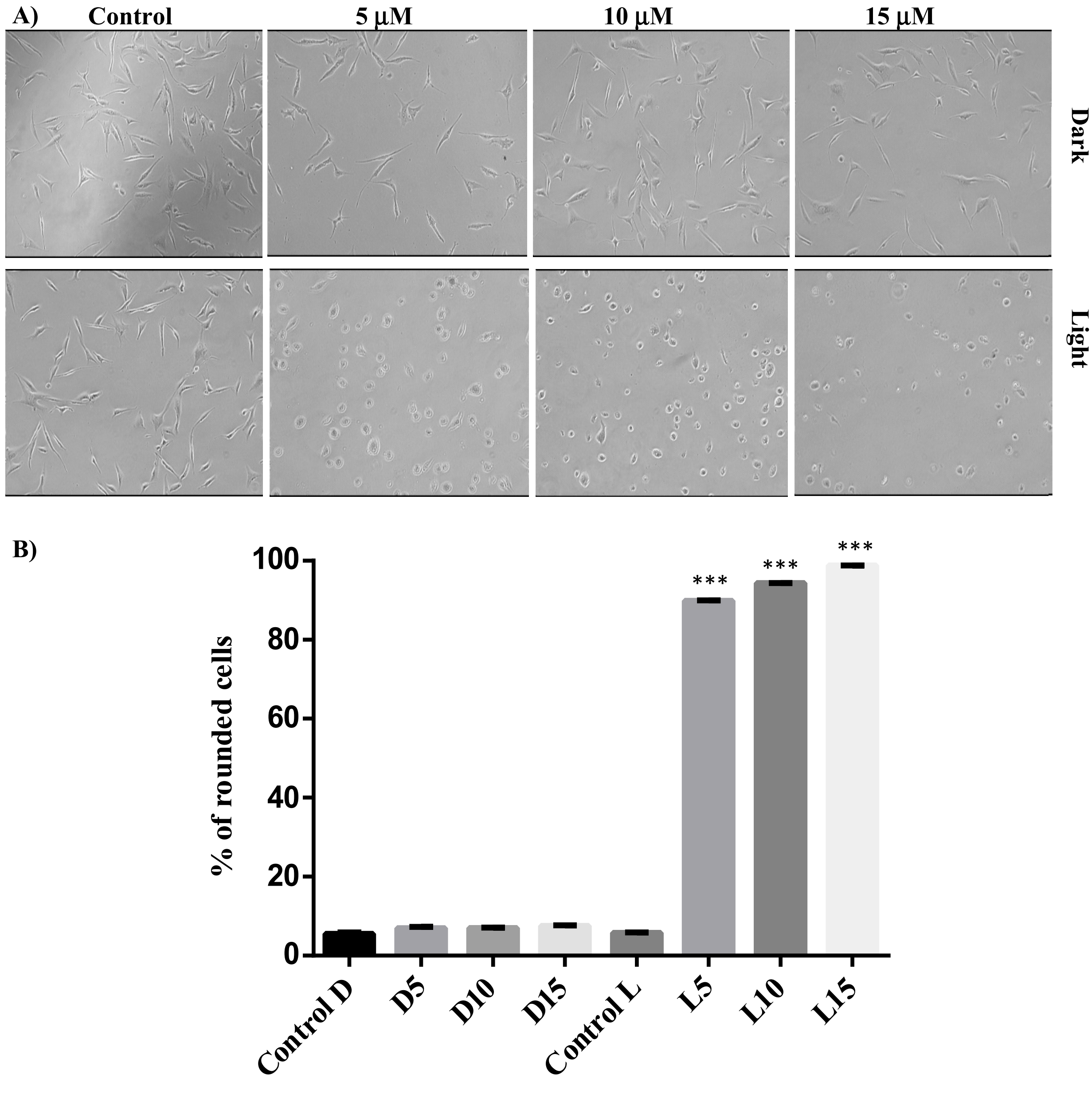

Figure S2. Morphological changes and cell rounding on A549 cells upon treatment with different concentration of complex 1 after 24h. (A). Representative images of A549 cells, as seen by an inverted light microscope. Cells were maintained in DMEM supplemented with 10% FBS and treated in the absence (control) or with 5, 10 and 15 μM of complex **1** in both dark and light conditions for 24h. A total of 10 images were evaluated for each condition and compared to control cells. **(B).** The percentage of cell rounding with and without treatment with complex **1** was measured using CASP software and the mean ± SEM was calculated from 3 different experiments. Results showed that treatment with complex 1 lead to significant increase in cell rounding, as indicated by *** ($p < 0.001$) when compared to control cells.

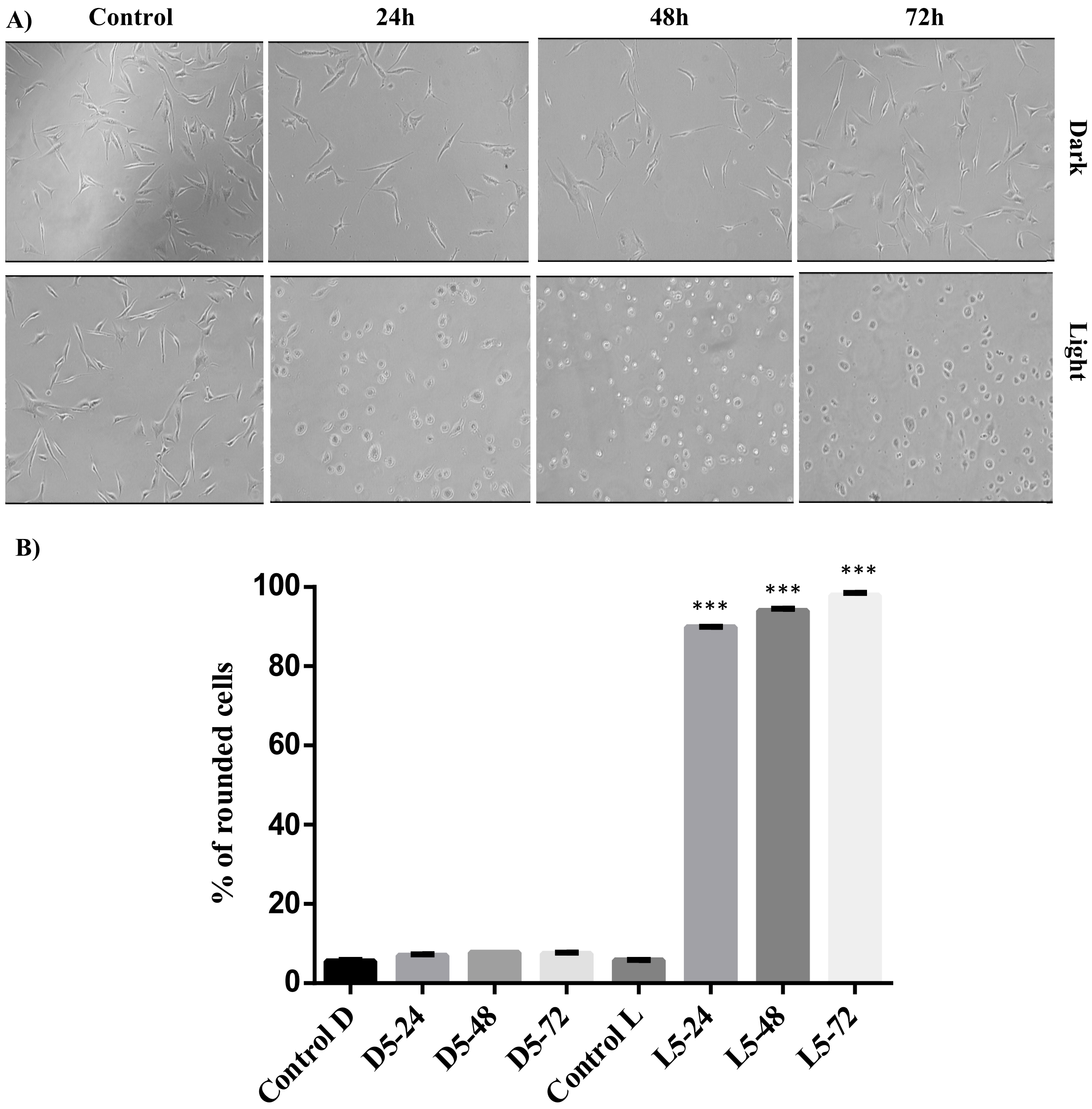

Figure S3. Morphological changes and cell rounding on A549 cells upon treatment with 5 µM of complex 1 for 24, 48 and 72h. (A). Representative images of A549 cells, as seen by an inverted light microscope. Cells were maintained in DMEM supplemented with 10% FBS and treated in the absence (control) or with 5 µM of complex **1** in both dark and light conditions for 24, 48 or 72 h. A total of 10 images were evaluated for each condition and compared to control cells. **(B).** The percentage of cell rounding with and without treatment with complex **1** was measured using CASP software and the mean ± SEM was calculated from 3 different experiments. Results showed that treatment with complex 1 lead to significant increase in cell rounding, as indicated by *** ($p < 0.001$) when compared to control cells.

Table S1. DNA damage in A549 cells exposed to 5 µM of complex 1 as measured by the comet assay after 24, 48 and 72h post exposure. The analysis was performed using CASP (Comet assay Software package).

	Incubation time (h)	Negative Control	Treated in Dark	Treated in Light	Positive Control
**Head Length**	24	129.35 ± 4.98	132.66 ± 0.93	101± 1.34*	125.8 ± 4.28*
	48	78 ± 4.92	87.55 ± 6.35	117.5 ± 7.49*	85.66 ± 8.96
	72	115 ± 5.95	116.56 ± 8.98	110 ± 1.28	79.23 ± 4.45*
**Tail Length**	24	9.98 ± 2.34	8.33 ± 2.19	48.33 ± 2.52*	59.6 ± 2.62*
	48	5.27 ± 3.68	7.64 ± 3.97	40.5 ± 2.62*	75.5 ± 5.32*
	72	11.6 ± 2.01	9.22 ± 2.25	58.67 ± 1.42*	70.31 ± 2.20*
**Comet Length**	24	139.33 ± 5.51	140.99 ± 4.78	149.33 ± 2.87*	185.4 ± 6.78*
	48	83.27 ± 5.11	95.19 ± 6.14	158 ± 6.46*	161.16 ± 12.11*
	72	126.60 ± 6.22	125.78 ± 8.91	168.67 ± 2.7*	149.54 ± 5.23*
**Head DNA content**	24	97.87 ± 3.41	97.04 ± 2.45	80.07 ± 0.68*	57.03 ± 0.86*
	48	98.07 ± 4.83	98.57 ± 3.45	72.48 ± 2.18*	49.1 ± 3.37*
	72	95.27 ± 3.4	97.08 ± 3.48	60.39 ± 3.6*	41.75 ± 1.94*
**Tail DNA content**	24	2.13 ± 3.41	2.96 ± 3.04	19.93 ± 3.68*	42.97 ± 3.86*
	48	1.93 ± 2.83	1.43 ± 2.45	27.52 ± 2.18*	50.90 ± 3.37*
	72	4.73 ± 2.4	2.92 ± 2.48	39.61 ± 4.6*	58.25 ± 1.94*
**Tail Moment**	24	0.21 ± 0.07	0.24 ± 0.06	9.63 ± 0.09*	25.61 ± 0.10*
	48	0.11 ± 0.1	0.10 ± 0.09	11.14 ± 0.5*	38.42 ± 0.18*
	72	0.54 ± 0.4	0.27 ± 0.5	23.24 ± 0.6*	40.95 ± 0.4*

The results of the comet assay were analyzed using one-way ANOVA on GraphPad Prism, and the differences between the negative control (untreated) cells and treated cells were determined. * indicates significant differences ($P<0.05$) between the different conditions. The mean ± SEM was calculated from 50 randomly selected images.

LC-MS/MS parameters

LC-MS/MS instrument parameters were previously reported [1]. Briefly, LC-MS/MS data were acquired using the Ultimate 3000 RSLC/TSQ Endura system from Thermo Fisher Scientific. Complex **1** (1 µL) was injected on a Hypersil GOLD C8 column maintained at 40 °C. An isocratic mobile phase was used, consisting of 5 % A (H_2O containing 0.1% Formic acid) and 95 % B (MeOH containing 0.1% Formic acid), at 0.5 mL min^{-1}. MS-MS spectra were then acquired in positive mode following sample injection into the heated ESI probe [sheath gas (2.71 L min^{-1}), aux gas (5.78 L min^{-1}), sweep gas (1.5 L min^{-1}), ion transfer tube temperature (325 °C), vaporizer temperature (200 °C), positive ion spray voltage (3700 V)]. LC-MS/MS conditions are listed in Table S2. Data were processed using the Xcalibur software from Thermo Fisher Scientific (v. 4.1).

Table S2. LC-MS/MS conditions used for the detection of Complex **1**, as previously described [1].

**Mobile Phase**<br>**Isocratic**<br>**5% A: H2O (0.1% Formic acid)**<br>**95% B: MeOH (0.1% Formic acid)**<br>**Flow rate (0.5 mL min^{-1})**<br>**Column oven set at 40 °C**	
**Precursor ion (m/z)**	373.2
**Product ions (m/z)**	157.1
	294.4
**Collision energy (V)**	20
**Retention time (min)**	0.45
**Mass mode**	H-ESI positive mode
**Scan type**	SRM[a]

[a]SRM, selected reaction monitoring

References

1. Mehanna, S.; Bodman-Smith, K.; Daher, C.F.; Khnayzer, R.S. Rapid Quantification of Ruthenium(II) Polypyridyl Anti-Cancer Drugs Using a Selective Ligand Dissociation LC-MS/MS Method. *Anal. Methods* **2020**, doi:10.1039/D0AY01250E.
